# Global Health’s Evolution and Search for Identity

**DOI:** 10.3201/eid3101.241026

**Published:** 2025-01

**Authors:** Kevin M. De Cock

**Affiliations:** Independent consultant (Nairobi, Kenya)

**Keywords:** *Keywords: global health*, *international health*, *tropical medicine*, *public health*, *Sustainable Development Goals*, *development assistance*, *decolonizing global health*

## Abstract

Despite earlier attempts to define global health, the discipline’s boundaries are unclear, its priorities defined more by funding from high-income countries from the Global North than by global health trends. Governance and resource allocation are challenged by movements such as decolonizing global health. Inherent contradictions within global health derive from its historical evolution from tropical medicine and international health, as well as recent trends in infectious diseases. Demographic, socioeconomic, and epidemiologic transitions, including the rise in noncommunicable diseases, have eroded the concept of a binary world of developed and developing countries. Competitive tension has emerged between aspirations for global health security and health equity. Dominant principles should focus on vulnerable populations, transnational challenges such as migration and climate change, appropriate prevention and care, and epidemic preparedness and response capacity. As the 2030 target date for the United Nations Sustainable Development Goals approaches, reconceptualization of global health is required, or the discipline risks losing identity and relevance.

Interest in global health has increased in the 21st Century, driven by new challenges to health security, as well as the need for intensified responses to older concerns such as HIV/AIDS (*1*). Epidemics and pandemics such as Ebola and COVID-19 attracted extensive media coverage and disrupted whole societies. Philanthropic and civil-society organizations increasingly influence policies and programs, universities have expanded global health research and teaching, and the authority of traditional players—governmental agencies and multilateral organizations—has been challenged. Social media’s relentless expansion has broadened awareness but also spread misinformation.

Despite this greater attention, the discipline of global health is difficult to define, its boundaries are uncertain, priority setting is unclear, and governance is controversial. Humpty Dumpty’s comment that a word can mean whatever he wanted it to mean could apply to the term global health (*2*). Some might argue this lack of clarity does not matter, provided quality work gets done; such imprecision, however, can negatively influence understanding and communication around health in the world, funding decisions, alignment between health needs and programs, and research prioritization. A discipline without definition cannot articulate a philosophic, technical, or moral basis and risks losing cohesion of its community and respect for its scientific value.

Defining a future vision for global health is timely as the world considers requirements after the COVID-19 pandemic and assesses progress toward the United Nations 2030 Sustainable Development Goals (*3*). Tension has emerged in the potentially competitive quests for global health equity and global health security; recent experience showed the Global North (high-income countries, mostly of the Northern Hemisphere) turning inward in response to global epidemics. This article examines the origins and meaning of the concept of global health, discusses some of its contradictions and inconsistencies, and proposes some considerations for the future.

## Defining Global Health

Today’s global health is essentially whatever the Global North characterizes as such. Global health currently focuses on diseases, mostly infectious, of the Global South (countries not defined as high-income, mostly in the Southern Hemisphere), including HIV/AIDS, malaria, and respiratory and diarrheal diseases of childhood. Most practitioners who consider global health their prime discipline are from the Global North, funded by northern governmental and nongovernmental agencies, universities, and philanthropic organizations (*4*). Professionals from the Global South rarely self-identify as global health specialists, and they do not lead research or implementation in the Global North. Priorities, funding, and activities are not well aligned with today’s global burden of disease and trends. Decisions about global health program and research funding are mostly made in the Global North, with limited involvement of the Global South. Today’s global health is not truly global, and it is neither representative nor democratic.

## Origins and History of Global Health

In an influential paper in 2009, Koplan et al. attempted to define global health (*5*). The discipline evolved from earlier approaches to health in the world, from tropical medicine and then international health. Geopolitical and health events over the late 20th Century drove the evolution toward global health, but ambiguity and inherent contradictions have remained.

Tropical medicine developed in the late 19th and early 20th Centuries in response to health needs of European colonizing powers and their constituents. Specialized schools were established in Liverpool, London, and Antwerp, focusing on infectious, predominantly parasitic, diseases related to the tropical environment. West Africa was called “the white man’s grave” because so many colonialists died from malaria and other epidemic-prone diseases. Control of sleeping sickness in the Democratic Republic of the Congo up to the time of independence illustrated the efficacy as well as the rigidity of some colonial interventions (*6*). Missionaries and militaries were players in the practice of tropical medicine. The United States, not a classic colonial power, contributed through efforts such as Walter Reed’s research on yellow fever and early programs supported by the Rockefeller Foundation (*7*).

Although tropical medicine has receded as a guiding concept (*8*), it spawned a modern movement focused on neglected tropical diseases, diseases that affect the poorest in the Global South (*9*). Contributions from the tropical medicine era continue to inform scientific understanding and modern infectious disease control. Traditional schools of tropical medicine identify themselves today as global health institutions but have retained their old names because of their brand value.

The second half of the 20th Century saw 2 trends, independence of the former European colonies and a reduced burden of infectious diseases, at least in the Global North. An overly optimistic interpretation was that infectious diseases were vanquished (*10*). Tropical medicine became an obscure interest, dominated by clinical parasitology. The early postindependence world was divided into developed and developing countries, with the former providing development aid to the latter.

International health emerged and focused on maternal and child health, nutrition, family planning, and population control for the developing world. Health activities were peripheral to development funding that emphasized agriculture and, to a lesser extent, education. Health ministries in the Global South were generally weak and of low visibility, especially compared with countries’ ministries of finance (*11*). The priority of the World Health Organization (WHO) was primary healthcare, as documented in 1978 in the Declaration of Alma Ata (*12*).

Advocacy and programs addressed disparities in maternal and child mortality (*13*). International health saw a binary world in which health assistance offered by rich countries to poor countries emphasized cost effectiveness; for such countries, a lower standard of interventions, prioritization of community health, and little support for complex individual care were tacitly accepted.

International health could nonetheless claim successes. The eradication of smallpox was achieved largely through collaboration between agencies with international reach (principally WHO, Centers for Disease Control and Preventioon, and US Agency for International Development) and countries of the South (*14*). Leadership from the Global North provided political and financial support for reproductive health, education and empowerment of women, and child survival (*15*,*16*). Mortality rates among children <5 years of age (under-5) and maternal deaths slowly reduced, despite enormous disparities; still today, sub-Saharan Africa and South Asia contribute more than half of all childhood deaths worldwide (*17*).

It was not traditional health challenges that promoted the concept of global health, but developments in other infectious diseases. Identification of Ebola virus in 1976 followed descriptions of Lassa and Marburg viruses in the 1960s (*18*–*20*). Recognition of AIDS in 1981 demonstrated the threat posed by emergence of unknown infections (*21*). The interaction between HIV and tuberculosis (TB) (*22*), along with the spread of multidrug-resistant TB, led WHO to declare TB a global emergency in 1993 (*23**,**24*). WHO established its first AIDS program in 1986 under the leadership of the late Jonathan Mann (*25*). The Special Programme on AIDS was later renamed the Global Programme on AIDS; its acronym, GPA, became synonymous with the world’s response to the AIDS pandemic and was probably the first entry of the term “global” into widespread public health use.

## Global Health in the Modern Era

Several influential documents were published in the early 1990s in the face of worrying infectious disease trends. The World Bank devoted its annual report in 1993 exclusively to health (*11*); it asserted that ill health was an impediment to the economic development of lower-income countries and that 3 diseases, AIDS, TB, and malaria, were disproportionately restricting development in sub-Saharan Africa. It also argued for increased investment in appropriate clinical services such as for trauma and obstetric care, a conceptual shift from international health’s general avoidance of curative or individual care.

The importance of emerging and reemerging infectious diseases was emphasized in publications from the Institute of Medicine (today the National Academy of Medicine of the National Academies of Science) (*26*) and CDC, including launch of a new scientific journal (*27*,*28*). Earlier opinions (*10*) that infectious diseases were no longer relevant were rejected as evidence mounted of new, newly recognized, resurgent, and drug-resistant infections. More than any other disease, HIV/AIDS drove development of the concept and practice of global health.

Demographic analyses over the 1990s showed that countries in East and southern Africa were experiencing major losses of life expectancy because of AIDS (*29*), raising concern that generalized HIV epidemics would engulf West Africa and the vast populations of Asia. The link between HIV and worsening TB trends was increasingly evident. The uncontrolled AIDS pandemic, its multisectoral impact, and skepticism about WHO’s effectiveness led to the creation of the Joint United Nations Programme on HIV/AIDS (UNAIDS), the first instance of a multilateral body established to address a single disease (*30*).

The 1996 International AIDS Conference in Vancouver, British Columbia, Canada, represented a watershed moment in science but also in advocacy. Combination antiretroviral therapy (ART) was shown to reverse immune deficiency and save lives (*31*); activists and other commentators immediately noted the inequity of drugs available in the Global North but not in the Global South, where the AIDS burden was highest. Patent protection, pharmaceutical profits, drug prices, generic medications, flexibilities under the TRIPS Agreement, and access to care became topics of passionate debate within health circles. Four years later, the biannual conference held in Durban, South Africa, gave many of the thousands of international delegates their first exposure to realities in Africa, against a backdrop of AIDS denialism by the country’s president and fierce and eloquent activism from civil society (*32*). AIDS now represented not only a health crisis but also a political one of international dimensions. Health had evolved from a development issue to a geopolitical concern.

## Increased Global Health Funding and Changing Epidemiology

In the early 21st Century, discussions of global health moved to the highest levels of political leadership (*30*,*33*). UN Secretary General Kofi Annan called for a war chest to combat disease in Africa. The UN-sponsored Millennium Development Goals defined 3 health goals: child survival; maternal mortality; and AIDS, TB, and malaria (*34*). The years 2002 and 2003 saw launch of the Global Fund, WHO’s 3x5 initiative (*35*), and the US President’s Emergency Plan for AIDS Relief (PEPFAR) (*36*), as well as the President’s Malaria Initiative (PMI) (*37*). Funding discussions were now about billions, not millions, of dollars for health assistance.

Development assistance for health, excluding funding for COVID-19, was almost $46 billion in 2021. Whereas this amount was vastly more than in previous decades, it still represented <1% of the world’s total health expenditures and less than one third of health spending in the poorest countries (*4*). Development assistance for health, which broadly corresponds to the Global North’s conceptualization of global health, is now a small proportion of current health spending overall. Most health funding in the Global South today comes from countries and their own populations.

Before COVID-19, approximately one third of assistance went to HIV/AIDS, TB, and malaria and another one third to maternal, neonatal, and child health. This funding has undoubtedly yielded results; the under-5 mortality rate, for example, is less than half what it was in 2000, and more than three quarters of all persons living with HIV are now accessing ART. Nonetheless, despite continued need in these areas, development assistance for health is not matched to the changing health trends of today’s world.

Infectious diseases are no longer the world’s leading cause of death; overall, three quarters of the ≈60 million deaths annually are from noncommunicable diseases (*38*). AIDS, TB, and malaria contribute <5% of global deaths. Inadequate attention is given in the Global South to structural interventions addressing risk factors such as tobacco use and overconsumption of salt and sugar. WHO has an ambitious agenda for tackling the noncommunicable disease pandemic, devoting high-level meetings at the United Nations to the topic (*39**,**40*); however, <2% of development assistance for health addresses that increasing burden in low- and middle-income countries (*4*).

## Reinterpreting Global Health

Health in the world has changed. The 21st Century has witnessed better understanding and lessening of the AIDS crisis; severe, widespread infectious disease epidemics, especially from different viruses such as Ebola, dengue, chikungunya, yellow fever, SARS, and others; the influenza (H1N1) and COVID-19 pandemics; improved child survival and life expectancy; and relentless increase in the burden of noncommunicable diseases (*41*). Conceptual shifts include increased recognition of interconnected global vulnerability to infectious diseases and other health shocks; emergence of new, transnational challenges to health, such as climate change; and realization that countries are more similar than different in our changing world.

Earlier division of the world into the “us and them” of developed and developing countries no longer holds. As the concept of global health was maturing, the World Bank introduced a stratification of national economies into high-income, middle-income, and low-income countries (with middle divided into upper- and lower-middle). In the last century, disparities in wealth were greatest between the Global North and Global South. Today, enormous disparities in wealth exist within countries, and most poor persons in the world live in countries no longer considered low income. Geopolitics also evolved after the Cold War; countries of the Global South are more active and independent on the world stage. Multilateral agencies established after World War II to deal with reconstruction or population displacement seem increasingly maladapted to changed realities.

Everywhere, health and demographic transitions are contributing to improved child survival, lower fertility, higher life expectancy, reduced infectious disease burden, and increased “lifestyle” diseases that result from socioeconomic developments and commercial forces. Although such transitions are unequally distributed and widely staggered in time, countries are essentially on the same demographic and health trajectories toward safer, longer, healthier, yet still finite lives (*42*). Such a synthesis necessarily overlooks stubborn disparities. Simplification should not obscure local or regional epidemiology such as persistent HIV epidemics in sub-Saharan Africa, stable malaria in specific settings, or high rates of TB in certain populations.

Two exceptions limit those generalizations. First, in the very poorest countries, infectious diseases remain disproportionately high, especially neonatal conditions, lower respiratory infections, diarrheal diseases, and malaria (*38*). Second, there are those countries that the Global Fund characterizes as challenging operating environments, threatened by conflict, mismanagement, or other manmade or natural disasters (*43*). In such contexts with failing or disrupted health systems, traditional health assistance and humanitarian support remain priorities, and health trends may stagnate or reverse.

## Global Health Beyond the Sustainable Development Goals

Our current situation is of health assistance that is mismatched to disease burden, combined with lurching, reactive funding to predictably unpredictable epidemics. Comprehensive discussion of overall global health priorities, irrespective of funding sources, is lacking. The memory and lingering consequences of COVID-19 have elevated the importance of political, technical, legal, and financial aspects of pandemic preparedness (*44*), but those factors constitute only one element of global health. Similarly, reinterpretation of global health should not preclude funding for prior, unfinished priorities.

The first requirement is clarity on philosophic principles and ambitions. Protection of human rights and recognition of vulnerability are fundamental. Social justice, a fair distribution of the benefits and burdens of society, and respect for human dignity must be guiding principles, all aiming to reduce disparities in health and well-being. Global health should focus on issues that are transnational, cross borders, affect multiple countries, and cannot be addressed by one nation alone. Containment of epidemic-prone infectious diseases, with all the requirements from diagnostic capacity to health commodities such as vaccines, is an example that combines the search for equity as well as security. Applied to both health and security, equity means the same outcomes irrespective of different investments required.

The evolving crises of climate change and migration offer other examples of transnational challenges. Noninfectious threats such as global warming or conflict-driven population displacement exemplify the unequal vulnerability of certain populations. Another area of preventable illness and deaths for global health is that related to violence and injuries, intentional as well as unintentional, extending from physical conflict to adverse road traffic and occupational events.

Global health must champion the needs of the disadvantaged, which includes the poor, the disabled, and the marginalized, such as sex workers, prisoners, persons with substance use disorders, the elderly, migrants, and other socially excluded groups. Global health is necessarily political, needing to address structural risk factors and social and economic determinants that drive ill health. Approaching such causes of the causes of disease may engender controversy that is best met head-on by commitment to basic principles, strong science, and clear communications.

Global health must act with the speed of relevance, greater than that often observed in traditional health diplomacy, and with a technical emphasis on implementation science. Health systems, national public health institutes, universal health coverage, and benefits and weaknesses of horizontal versus vertical interventions will remain topics of debate (*1*,*45*). Laboratory, diagnostic, data management, and analytic capacity are currently inadequate and unequal. Access to increasingly important advances in informatics, artificial intelligence, and genomics must be assured globally.

The essentials of global public health systems are relevant to all countries and populations. Defining frameworks assists in drawing boundaries for global health and identifying priorities. Potential approaches are to dissect health requirements by life stages; viewing health through a prism of development, security, and public health (*1*); or prioritizing topics relevant to the global community, rather than to just an individual country (Table).

**Table:** Essentials for global public health in a One Health approach*

Elements of public health
Resilient health systems, governance, and financing
Epidemiologic surveillance and response capacity
National public health institutes
Health research capacity
Expertise in public health law
Neonatal and child health services
Maternal health services
Clinical services
Sexual and reproductive health services
Control of infectious diseases, including One Health and vaccination services
Nutrition and food safety
Water, sanitation, and hygiene
Air quality
Migrant health services
Mental health services, including for substance use and addiction
Occupational safety and health
Injury prevention and control, including transportation safety
Environmental health
Health mitigation of climate change
General health promotion and education

*One Health is an integrated, unifying approach that aims to sustainably balance and optimize the health of people, animals and ecosystems. Source: World Health Organization. https://www.who.int/health-topics/one-health

A life-stage approach could accommodate demographic changes occurring throughout the world. Younger nations, for example, face a youth bulge requiring investment in youth-friendly services, prevention of injuries, and attention to mental illness, including substance use, that has its highest incidence in younger age groups. By contrast, countries with aging populations need to address challenges such as neurodegenerative conditions, frailty, multisystem disease, and need for social care.

The framework of development, security, and public health offers lenses through which to analyze global health. Nutrition, secure food supplies, and reproductive health services are core issues for development. Epidemic and pandemic response capacity, strengthening One Health approaches (*46*), and addressing migrant health are essential to security. Interventions mediating health effects of climate change or expanding access to preventive and treatment services for noncommunicable diseases promote public health worldwide. Re-envisioning global health must continue to address uncompleted objectives; millions of persons, for example, remain dependent on PEPFAR for access to lifesaving medications.

## Governance, Funding, and Historical Legacies

Any discussion of global health requires consideration of funding and governance. WHO remains the fulcrum for formulating global health policy, but the agency is often far removed from programmatic funding and field realities. The COVID-19 pandemic showed that what mattered most for pandemic containment was strength and resiliency of systems, national and local leadership, and social cohesion. Within such parameters, development assistance for health represents a small contribution to overall health requirements. If, like politics, all public health is local, all global health must be national.

Recent years have witnessed calls in the Global North for diversity, equity, and inclusion and emergence of sociopolitical movements such as Black Lives Matter and #MeToo. Appeals for decolonization of historic museums and statues have extended to development assistance and global health itself (*47*–*49*). Decolonizing also lacks a clear definition; interpretations range from total rejection of current geopolitical and economic systems to more modest shifts in decision-making authority.

The “decolonizing global health” movement links global health back to tropical medicine and its origins, including the fact that prestigious institutes were established with funds derived from colonialism and some of its abuses (*50*; D. Molyneux, unpub. data, lecture to the Liverpool Medical Institution, “The Liverpool School of Tropical Medicine: Role in the Development of Tropical Medicine”). Despite controversial aspects only clarified retrospectively, tropical medicine provided fundamental knowledge for today’s neglected tropical disease control efforts, parasitology, medical entomology, arbovirology, and much else. Assuring a just and better future influenced by necessary evaluations of a past we cannot change requires judgment.

Issues at stake today include global health leadership, priority setting, funding, and management of health research and programs in low- and middle-income settings. “Blowing everything up” risks overall disruption and interruption of care and programs for vulnerable populations. Understandably, taxpayers in the Global North will continue to expect accountability for use of development assistance funds. To some proponents, however, evolution toward greater fairness and inclusion seems slow and inadequate, and questions of power and trust must be addressed. If global health is to be global and inclusive, power cannot remain held exclusively in the Global North; broader trust lost during the COVID-19 pandemic needs to be regained.

## Conclusions

The discipline of global health is at an inflection point. It must refashion itself to ensure health security as well as delivery of services for the health trends of the coming decades, all in a spirit of solidarity and fairness. If not, global health risks eroding in relevance as a discipline and overarching health concept in an altered world. Such was the fate of tropical medicine. With the Sustainable Development Goals end date approaching, there is no room for delay.

Despite recent backlash against globalization and its unforeseen negative effects, the genie of globalized public health risk and information access is well out of the bottle. Change in global health conceptualization and implementation must be evaluated in real terms: disease and deaths averted, lives improved and prolonged. Our ultimate global health goals are security and also equity. Global health is what we as a global community can and must do for our world to be safe as well as healthy.
